# FimH Adhesin of Type 1 Fimbriae Is a Potent Inducer of Innate Antimicrobial Responses Which Requires TLR4 and Type 1 Interferon Signalling

**DOI:** 10.1371/journal.ppat.1000233

**Published:** 2008-12-05

**Authors:** Ali A. Ashkar, Karen L. Mossman, Brian K. Coombes, Carlton L. Gyles, Randy Mackenzie

**Affiliations:** 1 Centre for Gene Therapeutics, Department of Pathology and Molecular Medicine, McMaster University, Hamilton, Ontario, Canada; 2 Department of Biochemistry and Biomedical Sciences, McMaster University, Hamilton, Ontario, Canada; 3 Department of Pathobiology, Ontario Veterinary College, University of Guelph, Guelph, Ontario, Canada; University of Washington, United States of America

## Abstract

Components of bacteria have been shown to induce innate antiviral immunity via Toll-like receptors (TLRs). We have recently shown that FimH, the adhesin portion of type 1 fimbria, can induce the innate immune system via TLR4. Here we report that FimH induces potent *in vitro* and *in vivo* innate antimicrobial responses. FimH induced an innate antiviral state in murine macrophage and primary MEFs which was correlated with IFN-β production. Moreover, FimH induced the innate antiviral responses in cells from wild type, but not from MyD88^−/−^, Trif^−/−^, IFN−α/βR^−/−^ or IRF3^−/−^ mice. Vaginal delivery of FimH, but not LPS, completely protected wild type, but not MyD88^−/−^, IFN-α/βR^−/−^, IRF3^−/−^ or TLR4^−/−^ mice from subsequent genital HSV-2 challenge. The FimH-induced innate antiviral immunity correlated with the production of IFN-β, but not IFN-α or IFN-γ. To examine whether FimH plays a role in innate immune induction in the context of a natural infection, the innate immune responses to wild type uropathogenic *E. coli* (UPEC) and a FimH null mutant were examined in the urinary tract of C57Bl/6 (B6) mice and TLR4-deficient mice. While UPEC expressing FimH induced a robust polymorphonuclear response in B6, but not TLR4^−/−^ mice, mutant bacteria lacking FimH did not. In addition, the presence of TLR4 was essential for innate control of and protection against UPEC. Our results demonstrate that FimH is a potent inducer of innate antimicrobial responses and signals differently, from that of LPS, via TLR4 at mucosal surfaces. Our studies suggest that FimH can potentially be used as an innate microbicide against mucosal pathogens.

## Author Summary

The innate immune system is an evolutionarily conserved defence mechanism that protects the host from infection by microbes such as viruses, bacteria and fungi. Incoming pathogens are recognized by a set of evolutionary conserved receptors, including the Toll-like receptors (TLRs), that can be found on the surface of epithelial cells at the mucosal surface. We recently found that FimH, a specific adhesin located at the tip of type 1 fimbriae in uropathogenic *E. coli*, binds directly to TLR4. Here, we demonstrate the biological significance of this interaction. In the context of a natural infection, recognition of FimH by TLR4 is important for the host to mount an innate immune response against uropathogenic *E. coli*. Furthermore, we show that purified FimH protein induces a potent innate antiviral response, both in tissue culture and in animal models. This response is mediated predominantly by the production of type I interferon. Our results suggest that FimH is an excellent candidate for development as a microbicide against pathogen infection.

## Introduction

The innate immune system plays a crucial role in the early defence against microbial infections [1],[2],[3],[4],[5],[6]. A key aspect of the innate immune response is the synthesis and secretion of type I interferons (IFN) such as IFN-α and IFN-β. The innate immune system detects infections through germ-line encoded pattern recognition receptors [7], such as Toll-like receptors (TLRs). TLRs recognize conserved structures present in large groups of microorganisms, but not found in the host, called pathogen-associated molecular patterns (PAMPs) [8],[9],[10],[11],[12]. Thus far, 10 TLRs have been identified in mice and humans [13],[14],[15],[16], with each receptor recognizing a unique set of PAMPs [17],[18]. Examples of PAMPs include lipopolysaccharide (LPS, a ligand for TLR4), flagellin (a ligand for TLR5), double-stranded RNA (dsRNA, a ligand for TLR3), bacterial CpG DNA (a ligand for TLR9) and profillin (a ligand for TLR11). Upon ligand binding, TLRs initiate intracellular signalling through their cytoplasmic Toll/IL-1 (TIR) domain. These signalling pathways can be divided into common (MyD88-dependent) and specific (MyD88-independent) categories. TLR2, 5, 7–9 and 11 signalling is mainly MyD88-dependent, while TLR3 and 4 signalling is mediated through either MyD88 or Trif.

Recently, we and others have reported that CpG ODN/TLR9 signalling leads to the induction of potent innate protection against herpes simplex virus (HSV-2) infection both *in vivo* and *in vitro* [3],[19],[20],[21],[22],[23]. Local intravaginal (IVAG) delivery of CpG ODN or Poly I:C resulted in rapid proliferation and thickening of the vaginal epithelium and induction of a innate antiviral state that did not block virus entry but inhibited viral replication in vaginal epithelial cells. This TLR ligand-induced innate protection correlated with production of IFN-β, but not IFN-α, IFN-γ or TNF-α. Treatment of mice lacking the IFN-α/βR with CpG or Poly I:C did not provide innate antiviral protection against genital HSV-2 challenge compared to control mice. More recently, it has been shown that DC-derived IFNs are crucial for the innate antiviral activity of CpG in the genital tract [24].

Several PAMPs of bacterial origin including LPS, flagellin, peptidoglycan and bacterial DNA can activate the innate immune system via TLRs [18]. FimH, the adhesion portion of type 1 fimbriae produced by most *Enterobacteriaceae* including uropathogenic *E. coli*, is a conserved protein involved in bacterial attachment to mucosal epithelial cells [25],[26],[27]. Type I fimbriae have long been implicated in bacterial urinary tract infections in humans [25],[28],[29],[30],[31],[32],[33] and have been the focus of many attempts to generate a vaccine against pathogenic Gram-negative bacteria [26],[29],[34],[35],[36]. We have recently shown that recombinant FimH protein can activate the innate immune system through MyD88 and TLR4 in primary murine cells ([37] and un-published data). Little is known about the antiviral or antibacterial activity of FimH. Here we report that FimH is a potent inducer of innate antimicrobial responses. Our *in vitro* and *in vivo* experiments clearly show that FimH-induced innate antiviral immunity is associated with IFN-β production and requires MyD88, Trif, TLR4, IRF-3 and type I IFN signalling.

## Results

### FimH induces potent innate antiviral immunity against HSV-2 infection in RAW264.7 cells

**Figure 1:**
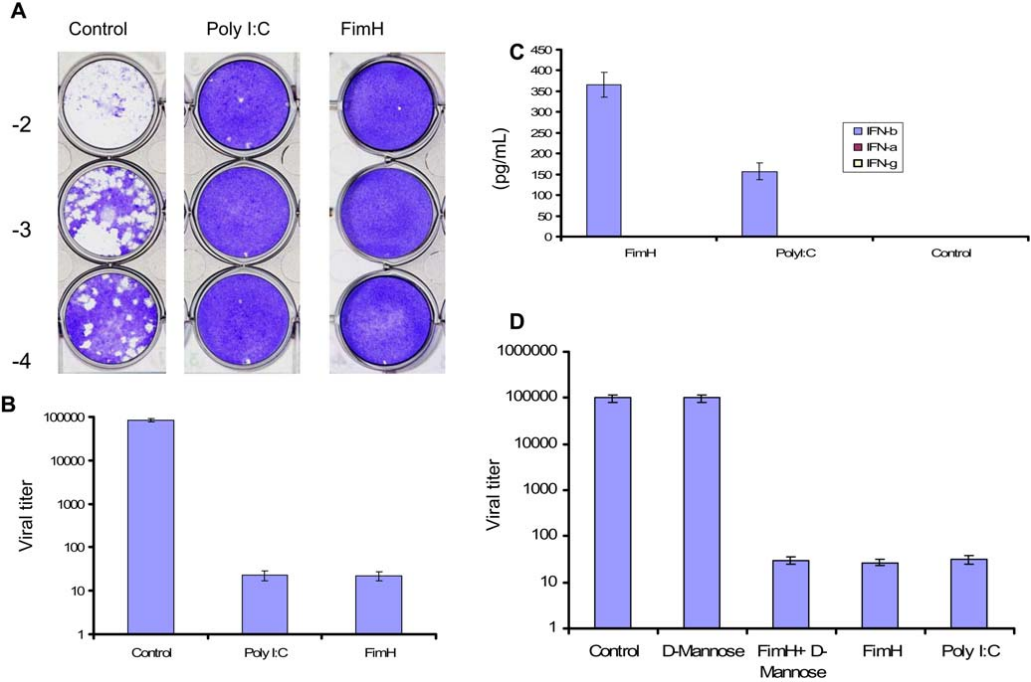
Treatment of RAW264.7 cells with FimH induces the production of IFN-β and significantly reduces HSV-2 replication. RAW264.7 cells were treated with either recombinant FimH (10 µg/mL), Poly I:C (10 µg/mL) or left untreated. Twenty-four hours post treatment cells were infected with HSV-2 with MOI of 0.1. Twenty hours post infection cells and the supernatant were collected and after 3 freeze/thaw cycles titered on Vero cells (A). Both FimH and Poly I:C treated cells had HSV-2 titers that were at least 2-3 logs lower compared to un-treated cells (A and B). Supernatants of treated cells were tested for IFN-α, IFN-β and IFN-γ (C). When FimH was incubated with alpha-D-Mannose (a standard blocker of binding to mannose) and then added to RAW264.7 cells, there was no inhibition of FimH innate antiviral activity (D).

We have previously reported that bacterial LPS and CpG DNA as well as Poly I:C can induce innate antiviral responses in RAW264.7 cells. More recently, we have shown that FimH can stimulate these cells and induce the production of TNF-α ([37] and un-published data). Here we first examined if FimH-mediated signalling resulted in antiviral activity. RAW264.7 cells treated with FimH had significantly lower HSV-2 titers compared to cells treated with PBS (**Fig.** **1A&B**). This innate protection correlated with the production of IFN-β, but not IFN-α or IFN-γ (**Fig. 1C**). It is well documented that FimH binds to alpha-D-mannosides [38],[39],[40],[41]. We then examined if the mannose binding domain of FimH is involved in the signaling. Incubation of FimH with D-mannose had no effect on FimH-induced innate antiviral response (**Fig.** **1D**).

### FimH induces strong innate antiviral responses (type I IFNs) in MEFs from B6, but not from MyD88^−/−^, Trif^−/−^, IFN-α/βR^−/−^ or IRF-3^−/−^ mice

**Figure 2:**
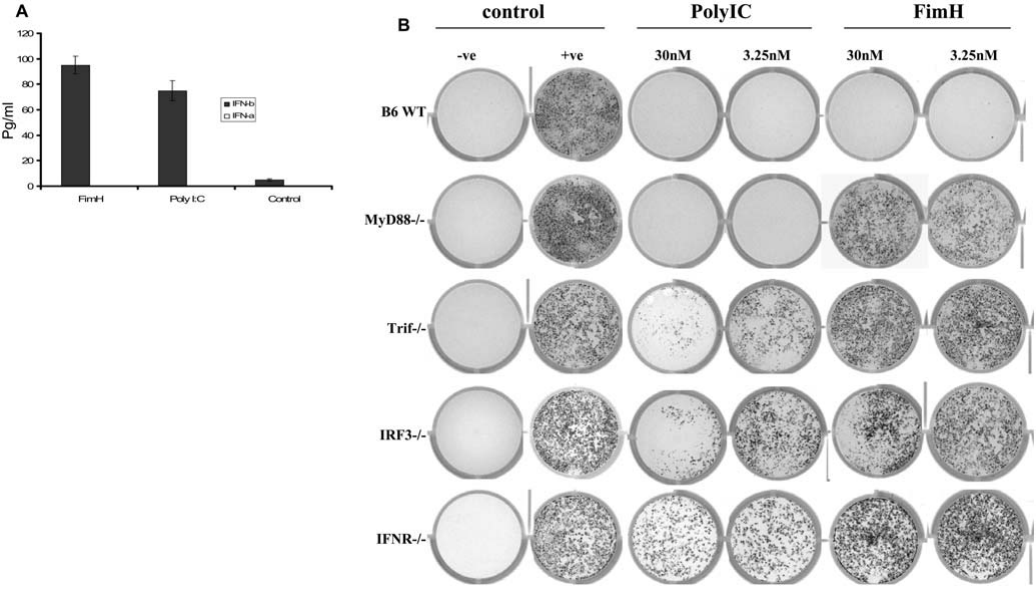
FimH induces strong innate antiviral responses in B6, but not MyD88^−/−^, Trif^−/−^, IRF-3^−/−^ or IFN-Rα/β ^−/−^MEFs. B6 MEFs were split at passage 3 and then treated with either FimH (10 µg/ml), Poly I:C (10 µg/ml) or left untreated for 24 hours. The supernatants were removed and were used to measure levels of IFN-α and IFN-β. Both FimH and Poly I:C treatment induced significant levels of IFN-β, but not IFN-α, in B6 MEFs (A). In a next series of experiments B6, IRF-3^−/−^, MyD88^−/−^, Trif^−/−^ or IFNα/βR^−/−^ MEFs, at passage three, were seeded into 12-well plates, 24 hours prior to treatment. Serial dilutions of Poly I:C or FimH were made in complete 10% alpha-MEM media, then 500 µl were added directly to the MEFs and cells were allowed to incubate at 37°C for 24 hours. Twenty-four hours post treatment the supernatants were aspirated and stored at −20°C for future use. Then 200 µl of VSV-GFP (3.18×10^4^ pfu/ml) in 0% alpha-MEM were added to each well and then cells were incubated for 1 hour at 37°C with rocking every 10 minutes. After 1 hour, VSV-GFP inoculums were aspirated from each well and cells were overlayed with 1 ml of 10% alpha-MEM /1% methylcellulose and incubated for 24 hours at 37°C. After 24 hours, VSV-GFP plaques were visualized using a Typhoon phosphoimager. (B) Represents photomicrographs of GFP expression in MEFs treated with FimH, Poly I;C or un-treated controls. In B6 MEFs FimH as well as Poly I:C treatment provide complete protection against VSV compared to controls. FimH failed to provide protection in MyD88^−/−^, Trif^−/−^ , IFNα/βR^−/−^ or IRF-3^−/−^ MEFs.

**Figure 3:**
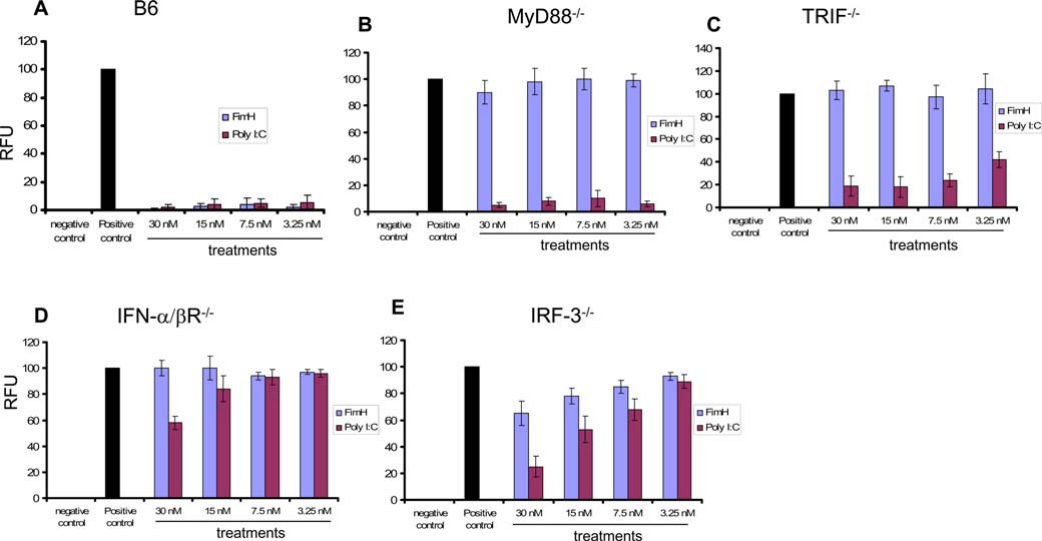
FimH induces strong innate antiviral responses in B6, but not MyD88^−/−^, Trif^−/−^, IRF-3^−/−^ or IFN-Rα/β ^−/−^MEFs. Figure 3 shows mean GFP florescent intensity of MEFs treated with different concentrations of FimH or Poly I:C relative to untreated VSV-GFP infected cells (RFU). Values from control un-infected wells were used as background. Values from infected, untreated, wells (positive controls) were set at 100%. B6 MEFs treated with FimH or Poly I:C had significantly lower infections (A). MyD88^−/−^, Trif^−/−^, IFNα/βR^−/−^ and IRF-3^−/−^ MEFs treated with FimH showed no significant reduction in relative GFP fluorescence compared to untreated positive controls (B–E). Poly I:C treatment provided complete protection against VSV in My D88^−/−^, but not Trif^−/−^, IFNα/βR^−/−^ or IRF-3^−/−^ MEFs.

Recently we have shown that FimH signalling required TLR4 and MyD88 pathway in murine macrophages [37]. It is well documented that Poly I:C induces strong antiviral responses in mouse embryonic fibroblasts (MEFs) as measured by a standard VSV plaque reduction assay. We first examined whether FimH could induce the production of type 1 IFNs, resulting in an innate antiviral state in B6 MEFs. Interestingly, FimH induced similar levels of IFN-β, but not IFN-α, in B6 MEFs compared to those treated with Poly I:C (**Fig. 2A**) and provided complete protection against VSV challenge (**Fig. 2B** **&** **3**). B6 MEFs treated with FimH also showed little or no VSV-GFP replication, as detected by GFP fluorescence, when compared to untreated MEFs (**Fig. 3A**). We then examined whether FimH-induced innate antiviral responses were MyD88, Trif and/or type I IFN dependent. FimH failed to provide protection against VSV challenge in MEFs deficient in either the MyD88 or Trif adaptors, whereas Poly I:C provided complete protection in MEFs lacking MyD88 and partial protection in MEFs lacking Trif (**Fig. 2B** **,** **3B,C**). To further investigate whether type 1 IFNs, particularly IFN-β, were involved in FimH-induced innate antiviral immunity, MEFs from IFN-α/βR^−/−^ or IRF-3^−/−^ mice were treated with FimH or Poly I:C and then challenged with VSV. FimH failed to induce an innate antiviral state in the absence of either IRF-3 or type 1 IFN signalling (**Fig. 2B** **,** **3D,E**). Poly I:C induced only moderate protection at 30 nM or 15 nM, but no significant differences in VSV-GFP fluorescence was observed in MEFs at lower concentrations compared to untreated MEFs (**Fig.** **2A,B** **,** **3D,E**).

### Vaginal delivery of recombinant FimH, but not LPS, protects normal B6 mice from subsequent IVAG HSV-2 challenge

**Figure 4:**
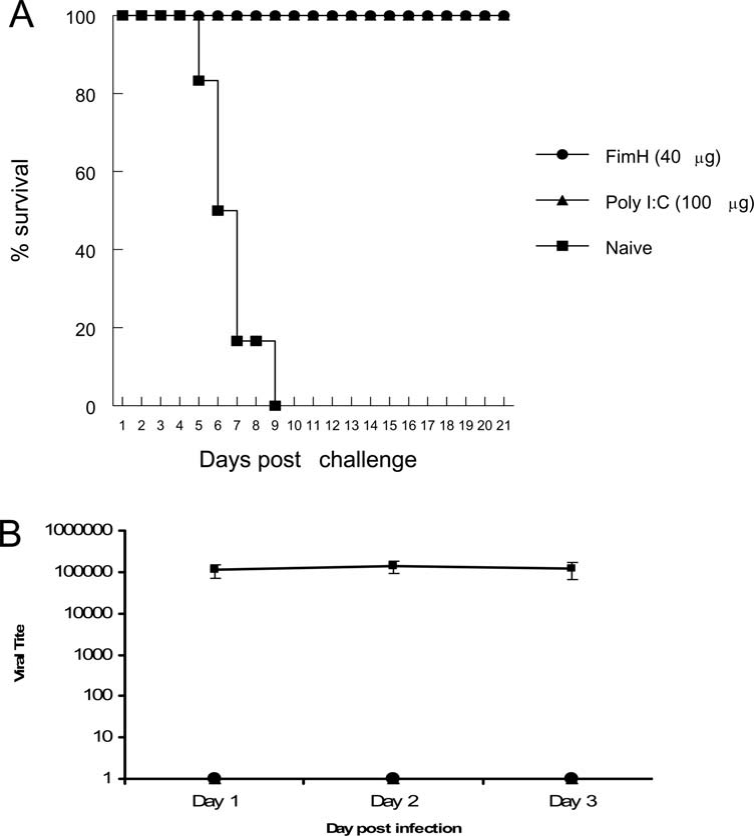
Local delivery of FimH induces potent innate antiviral immunity against IVAG HSV-2 challenge. Six- to eight-week-old B6 mice were treated with Depo-Provera to synchronize their susceptibility. Four days later, mice were treated locally with FimH or Poly I:C. Twenty-four hours post treatment mice were challenged IVAG with a lethal dose of HSV-2 (10^4^ pfu). Challenged mice were monitored daily for genital pathology, survival and vaginal virus titers. Mice that received FimH or Poly I:C were protected against IVAG HSV-2 challenge (A) and showed no HSV-2 titers in the genital washes compared to naïve mice (B).

We have reported that the mucosal delivery of some TLR ligands/agonists can induce an innate antiviral state and provide complete protection against subsequent IVAG HSV-2 challenge [3],[19],[22]. To examine if local delivery of FimH can provide innate protection against subsequent IVAG HSV-2 challenge, FimH was administered intravaginally to mice and then mice were challenged with lethal doses of HSV-2 24 hours following this treatment. FimH provided 100% protection against IVAG HSV-2 challenge compared to control mice (**Fig.** **4A**). Moreover, HSV-2 virus particles were not present in the vaginal washes from FimH-treated mice compared to control mice (**Fig.** **4B**).

**Figure 5:**
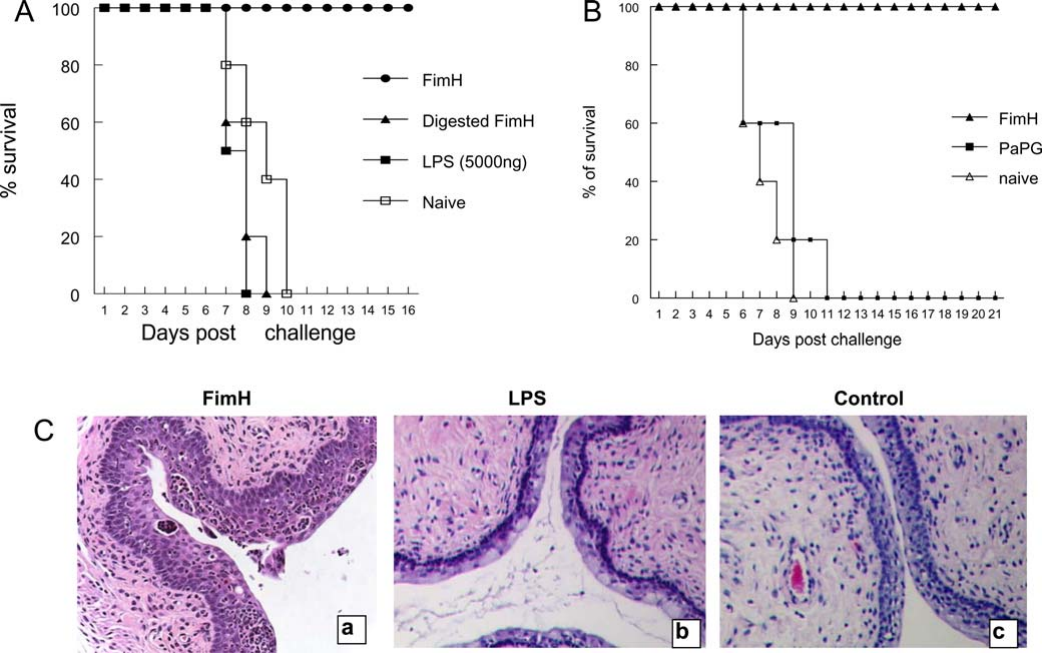
Induction of innate antiviral immunity by FimH at the genital mucosa is independent of LPS or other bacterial contaminants. Six- to eight-week-old B6 (N = 5 per group) mice were treated with Depo-Provera to synchronize their susceptibility to IVAG HSV-2 challenge. Four days later, mice were treated locally with intact FimH, digested and/or head-inactivated FimH or LPS. Another group of mice were treated with FimH, PapG or left untreated. Twenty-four hours post treatment mice were challenged IVAG with a lethal dose of HSV-2 (10^4^ pfu). Challenged mice were monitored daily for genital pathology, survival and vaginal virus titer. (A) Mice that received FimH (•) were protected against IVAG HSV-2 challenge compared to digested FimH (▴), LPS (▪) or naïve (□) mice. Treatment of mice with PaPG did not provide any protection against IVAG HSV-2 challenge (B). Figure 5C shows photomicrograph of vaginal tissue cut in cross section and stained with H&E.

To further verify that the innate antiviral protection in FimH-treated mice was due to the direct effects of FimH protein, but not LPS or other bacterial contaminants, we performed three experiments: 1) mice were treated with 5000 ng of LPS and then challenged with HSV-2; 2) mice were treated with protease-digested FimH (complete digestion was confirmed by gel electrophoresis) and then challenged with HSV-2; 3) mice were treated with either FimH or another component of bacterial pilin (PapG), which were expressed, purified and prepared in the same manner. As shown in **Figure 5A** neither LPS nor digested FimH protected mice against subsequent challenge with IVAG HSV-2. Mice treated with PapG were also not protected against IVAG HSV-2 challenge (**Fig. 5B**). However, both FimH and PapG were prepared identically and the preparations had similar levels of LPS. Interestingly, FimH, but not LPS, induced dramatic morphological changes in the genital mucosa, including thickening of the vaginal epithelium and recruitment of polymorphonuclear cells (PMNs) (**Fig. 5C**).

### MyD88 and IFN-β signalling as well as TLR4 are required for FimH-induced innate protection against genital HSV-2 infection

**Figure 6:**
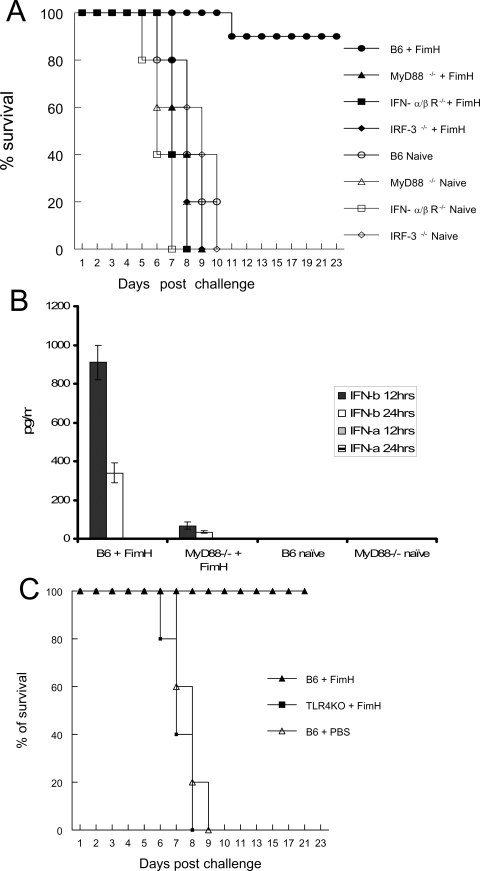
TLR4 and Type 1 IFN signalling, particularly IFN-β, are required for FimH-induced innate protection against HSV-2 challenge. Six- to eight-week-old female B6, IRF-3^−/−^, MyD88^−/−^ or IFNα/βR^−/−^ were treated with Depo-Provera. Four days later, mice were treated with either FimH (40 µg/mouse) or left untreated. Twenty-four hours post treatment mice were challenged with a lethal dose IVAG of HSV-2 (10^4^ pfu). Challenged mice were monitored daily for genital pathology, survival and vaginal virus titers. (A) Only B6 mice that received FimH (•) showed 90% protection against IVAG HSV-2 challenge compared to naive B6 (○) or FimH treated MyD88^−/−^ (▴), IFNα/βR^−/−^ (▪) or IRF-3^−/−^ (♦) mice. Figure 11b shows that local delivery of FimH induces production of significant levels IFN-β in B6, but not MyD88^−/−^ mice. Six- to eight-week-old female B6 or MyD88^−/−^ mice were treated with either FimH (40 µg/mouse) or left untreated. (B) Vaginal washes were collected at 12 and 24 hours post treatment and were used to measure levels of IFN-β and IFN-α using ELISA. Treatment of MyD88^−/−^ mice induced significantly (p<0.001) lower levels of IFN-β compared to B6 controls. (C) 6–8 week-old B6 mice TLR4^−/−^ mice were treated with FimH and then challenged with IVAG HSV-2 as described above. Local delivery of FimH failed to provide innate protection against IVAG HSV-2 challenge in TLR4^−/−^ mice.

Our *in vitro* observations show that FimH signalling requires TLR4, MyD88 and type 1 IFN signalling to induce innate antiviral activity. Thus, we examined whether vaginal delivery of FimH could protect mice lacking these innate factors against genital HSV-2 infection. While FimH provided nearly complete protection in B6 mice, there was no protection against IVAG HSV-2 challenge in FimH-treated MyD88^−/−^ mice (**Fig. 6A**). We also examined if type I IFNs, particularly IFN-β, were involved in the FimH-induced innate antiviral immunity *in vivo*. Vaginal delivery of FimH and Poly I:C to IFN-α/βR^−/−^ and IRF-3^−/−^ mice failed to protect them against IVAG HSV-2 challenge compared to control mice (**Fig.** **6A**). This innate protection strongly correlated with the production of IFN-β, but not IFN-α, levels in the vaginal washes (**Fig. 6B**). To ensure that the ELISA kit could detect naturally produced IFN-α, we used supernatants from BM-DCs treated with Poly I:C or CpG ODN and were able to detect high levels of mIFN-α (Figure S1). Finally, to confirm that FimH signals via TLR4, B6 and TLR4^−/−^ mice were treated with FimH and then challenged with IVAG HSV-2. FimH failed to protect TLR4^−/−^ mice but completely protected B6 mice against genital HSV-2 challenge (**Fig. 6C**).

### TLR4 is essential for innate response to FimH-expressing bacteria

**Figure 7:**
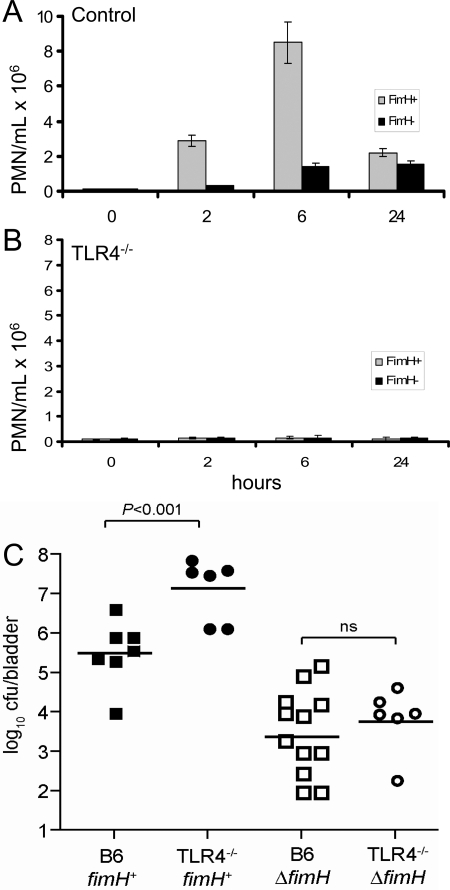
TLR4 is required for innate response to FimH+ UPEC. (A) Polymorphonuclear response to FimH+ and FimH- UPEC. B6 and TLR4^−/−^ female mice, aged 8–10 weeks, were used for the experiments. For infections, 0.1 ml (10^8^ cfu) of FimH+ or FimH− UPEC was injected into the bladder of anesthetized mice using a soft catheter (0.7 mm) placed in the urethra. Urine was collected from uninfected and infected mice and the cfu was enumerated. (N = 5 per time point). Polymorphonuclear leukocytes in urine were quantified using a haemocytometer. (B) UPEC colonization of the bladder. B6 and TLR4^−/−^ mice were infected with either wild type or *fimH^−^* UPEC as described in Materials and Methods. Twenty-four hours after infection, the bladders were removed, homogenized and the bacterial load was enumerated (C). Each data point represents one infected animal and data shown is from three independent experiments. Horizontal lines within each scatter of data represent the geometric mean. B6 vs TLR^−/−^ infected with wild type UPEC (*P*<0.001, ANOVA); ns, not significant.

FimH plays an important role in the pathogenicity of uropathogenic *E. coli* (UPEC) [26]. To examine whether FimH plays a role in innate immune induction in the context of a natural infection, we measured PMN leukocyte recruitment to the urinary tract in B6 mice and TLR4-deficient mice following infection with wild type UPEC and a fimH null mutant. In B6 mice, UPEC expressing FimH induced a rapid PMN response whereas mutant bacteria lacking FimH did not (**Fig. 7A**). This FimH-induced PMN influx required TLR4, since the cellular influx was blocked in TLR4-deficient mice (**Fig. 7B**). The bacterial load was enumerated in the bladder 24 h after infection. TLR4 was required for control of infection in the bladder, as TLR4^−/−^ mice had ∼1.5-log more wild type bacteria in the bladder at 24 h compared to B6 mice (**Fig. 7C**), which correlated with a loss of PMN recruitment in the TLR4^−/−^ mice. Deletion of fimH resulted in decreased colonization of the bladder but this decrease was not overcome in a TLR4^−/−^ background, confirming that while FimH is important for UPEC colonization of the bladder [33], FimH-independent signaling through TLR4 is not likely a major contributor to infection control in B6 mice.

## Discussion

We have demonstrated here that FimH can induce a potent innate antiviral state, both *in vitr*o and *in vivo*. Pre-treatment of MEFs from B6 mice, but not MyD88^−/−^, Trif^−/−^, IRF-3^−/−^ or IFN-α/βR^−/−^ mice, with FimH conferred protective antiviral responses. Mucosal delivery of FimH, but not LPS, provided complete protection against IVAG HSV-2 challenge in B6 mice while it failed to provide any protection in MyD88^−/−^ mice. Moreover, the FimH-induced innate antiviral immunity was associated with the induction of IFN-β in the genital tract and required TLR4 and IRF-3. To evaluate the biological significance of FimH in host pathogen interaction, we have examined the host innate response in FimH knockout uropathogenic E.coli in the presence and absence of TLR4. TLR4 was required for the induction of innate immune responses against UPEC.

We have shown that FimH requires TLR4 and MyD88 to activate murine primary macrophages. In addition we have also reported that dsRNA and CpG ODN confer protection against HSV-2, both *in vitro* and *in vivo* [3],[19],[22]. Similar to Poly I:C and CpG, the FimH-induced innate antiviral state correlates with IFN-β, but not IFN-α or IFN-γ production. Although we have observed high levels of TNF-α, it is unlikely that the FimH induced innate antiviral activity is mediated via TNF-α. Previous work showed that treatment of RAW264.7 cells with TNF-α cannot protect them against HSV-2 infection [42]. FimH also induces significant levels of NO production. Both IFN-β and NO are able to block HSV-2 replication [1],[2],[43],[44],[45].

We have observed induction of a strong innate antiviral state by FimH that correlated with the production of IFN-β, and required TLR4. It was first important to establish whether, in addition to TLR4, FimH binding to its natural receptor, mannose, is essential for induction of innate antiviral activity. It is well documented that FimH adhesin of uropathogenic *E. coli* type 1 fimbriae bind to mannose on epithelial cells. Blocking the mannose-binding portion of FimH with D-mannose had no effect on the FimH-induced innate antiviral activity. This suggested that FimH may bind to TLR4 independent of mannose to induce antiviral responses. We were unable to detect any IFN-α from FimH treated RAW264.7 or B6 MEFS by ELISA. This suggested that IFN-β is the key factor in the FimH-induced innate antiviral state. Given the importance of the transcription factor IRF3 in the production of IFN-β in fibroblast and epithelial cells, FimH also failed to induce IFN-β production from IRF-3^−/−^ MEFs and did not protect these cells against VSV. Taken together, these data indicate that FimH signals through IRF3 in the induction of an innate antiviral response.

We and others have shown that mucosal delivery of TLR ligands protect mice against subsequent IVAG HSV-2 challenge [3],[19],[20],[21],[22],[46]. More recently, we have found that the TLR ligand-induced innate antiviral responses against IVAG HSV-2 strongly correlate with the production of IFN-β, but not IFN-α [47]. Our data show that FimH activity of FimH against genital HSV-2 challenge requires MyD88^−/−^, IRF-3^−/−^ IFN-α/βR^−/−^ and TLR4^−/−^ mice did not provide any protection against subsequent IVAG HSV-2 challenge compared to B6 control. FimH also induced significantly lower levels of IFN-β in MyD88^−/−^ mice while IFN-β was not detectable in IRF-3^−/−^ mice compared to B6 control mice. This clearly suggested that FimH-induced innate antiviral activity against IVAG HSV-2 is mediated via TLR4, MyD88 and type 1 IFNs, particularly IFN-β.

Since we purified recombinant FimH from bacteria, it was essential to confirm that the antiviral activity seen with the purified FimH was not due to LPS contamination and/or other possible minor proteins. Our *in vitro* and *in vivo* data clearly showed that the antiviral activity of FimH was not due to contamination with LPS. We have used all possible controls to confirm that FimH was responsible for the innate antiviral responses. First, control samples from bacteria that contained empty vector and processed in exactly the same manner as FimH had no antiviral activity assuring that the antiviral response seen with FimH is not due to low levels of LPS contamination. Second, both enzymatic digestion and heat inactivation of FimH protein significantly abrogated the activity of FimH protein *in vitro* and *in vivo*. To also confirm that FimH, but not LPS, is responsible for *in vivo* innate antiviral responses, we performed several experiments. First; local delivery of LPS or digested/heat-inactivated FimH protein gave no protection against subsequent IVAG HSV-2 challenge in B6 mice compared to treatment with intact FimH protein. Second; local delivery of recombinant PapG protein, another adhesin of fimbriated bacteria which was prepared exactly with the same protocol as FimH, gave no protection against subsequent IVAG HSV-2 challenge in B6 mice compared to FimH. However, PapG protein had the same levels of LPS compared to FimH. In addition, our *in vitro* experiments clearly showed that low levels of LPS present in our samples cannot provide any protection against viral infections. In addition we have shown that FimH can directly bind TLR4 ([37] and un-published data). Furthermore, while FimH induced dramatic changes in genital mucosa, there was no difference in the histomorphology of the genital mucosa from LPS- or PBS-treated mice. More importantly, our recent data indicates that FimH signals in cells unresponsive to LPS ([37] and un-published data).

It is well known that FimH play an import role in attachment of bacteria to epithelial cells and contributes to pathogenecity of UPEC. Our data show that TLR4 is involved in FimH signalling with epithelial cells. FimH-expressing UPEC were able to induce recruitment of PMNs to the urinary tract of wild type mice, while isogenic bacteria lacking FimH did not. Interestingly, this response is controlled by TLR4 expression and is abolished when we used FimH^−^ UPEC, even in the presence of TLR4. These data are similar to the PMN response seen following infection with a type I fimbriated derivative of non-adhesive *E. coli* [48]. Because these UPEC strains share expression of another TLR4-activating PAMP (LPS), these data suggest that the dominant innate immune-activating PAMP on uropathogengic *E. coli* may in fact be FimH. In support of this, deletion of *fimH* resulted in decreased colonization of the bladder as reported previously [33] but the level of colonization by *fimH^−^* UPEC was similar in a B6 and TLR4^−/−^ background. These data suggest that FimH-independent signaling through TLR4 is not likely a major contributor to infection control in B6 mice.

This is the first report to show that FimH has potent innate antiviral activity which also requires TLR4, MyD88, Trif, IRF-3 and type 1 IFNs, particularly IFN-β. So far, TLR ligands such as dsRNA, ssRNA, and CpG DNA have been associated with the induction of innate antiviral immunity. Protein ligands of TLRs have not been associated with the induction of innate antiviral immunity. Here, however, we demonstrate that FimH, but not LPS, mediates innate antiviral activity at the genital mucosa. It is of particular interest that both TLR5 and TLR11 ligands signal via MyD88, whereas FimH protein signals through both MyD88 and Trif, leading to activation of the IRF-3 pathway. Results from this study may provide the basis for a novel mucosal innate microbicide for a vast variety of mucosal viral infections such as HSV-2, HIV-1 or other sexually transmitted infections.

## Materials and Methods

### Mice

Female C57BL/6, 129SVPasCrl mice, 8–12 weeks old, were purchased from Charles River Laboratory (Quebec, Canada). TLR4^−/−^ mice were purchased from Jackson laboratory (Bar Harbor, USA). Breeding pairs of IFNα/βR^−/−^ were kindly provided by Rolf M. Zinkernagel (Zürich, Switzerland). Breeding pairs of IRF-3^−/−^, MyD88^−/−^ and Trif^−/−^ mice were kindly provided by Dr. T. Taniguchi (via Dr. T. Moran), Dr. S. Akira (via Dr. D. Golenbock) and Dr. B. Beutler, respectively. All mice were housed in level B rooms which followed a 12 hour day and 12 hour night schedule, and were maintained under standard temperature controlled conditions.

### Cells, viruses and reagents

RAW264.7, HEL fibroblasts and BJ fibroblasts cells were purchased from ATCC. B6, IRF-3^−/−^, MyD88^−/−^, Trif^−/−^ and IFNα/βR^−/−^ murine embryonic fibroblasts (MEFs) were prepared from gestation day 13.5 in α-MEM with 20% FBS and weaned then grown in 10% α-MEM for experiments. 293, 293-hTLR4 and 293-hTLR4-CD14/MD2 cells were purchased from InvivoGen and maintained in 10% DMEM supplemented with 10ug/mL blasticidin (293-hTLR4) or 10ug/mL blasticidin and 50ug/mL HygroGold (293-hTLR4-CD14/MD2). HSV-2 strain 333 was grown and titred as previously described [49]. VSV expressing GFP was kindly provided by Dr. Brian Lichty (McMaster University, Hamilton, ON). GM-CSF was purchased from R&D. α-D-manosidase, LPS (L26-54) and Poly I:C were purchased from Sigma (Oakville, ON, Canada). Depo-Provera was purchased from Upjohn (Don Mills, ON, Canada).

### Purification of FimH

The *fimH* gene from avian pathogenic *E. coli* strain EC99 (O78) was cloned into pQE-30 and expressed in BL-21 competent *E. coli*. FimH expression and purification were performed as previously described [50]. Briefly, Protein expression was induced by adding 1M IPTG to a final concentration of 1 mM and induction continued for a period of 5 hours. Bacterial pellets were lysed and protein isolation continued under denaturing conditions utilizing Ni-NTA affinity chromatography. Isolated protein fractions were then dialyzed in a 10-kDa Slidlyzer dialysis cassette against PBS. The LPS contraction in the purified FimH protein was determined using Limulus Amebocyte Lysate LPS detection kit according to the manufacturer's protocol.

### Treatment of RAW264.7 cells and MEFs with ligands

RAW264.7 cells were treated with FimH (10 µg/ml), Poly I:C (10 µg/ml) or left un-treated. Twenty-four hours post treatment the supernatants were collected and stored at -20°C for further study. The cells were then infected with HSV-2, MOI of 0.1. Twenty to twenty-two hours post infection the cells and supernatants were collected for HSV-2 titration on Vero cells. Passage 3 MEFs from B6, IRF-3^−/−^, MyD88^−/−^, Trif^−/−^ and IFNα/βR^−/−^ mice were split into 12-well plates and then treated with various concentrations of FimH, Poly I:C or LPS or left un-treated. Twenty-four hours post treatment, MEFs were infected with VSV-GFP. Levels of GFP fluorescence were visualized and quantified using a Typhoon™ scanner (GE Healthcare) 24 hours post-infection.

### Local delivery of TLR ligands, genital HSV-2 inoculation and vaginal virus titration

B6, TLR4^−/−^,IRF-3^−/−^, MyD88^−/−^ and IFNα/βR^−/−^ mice, 6–8 weeks old, were subcutaneously (sc) injected with 2 mg of progesterone/mouse (Depo-Provera). Four days later the mice were anaesthetized and treated vaginally with FimH (40 µg/mouse) or Poly I:C (100 µg/mouse). Twenty-four hours after treatment the mice were anesthetised, placed on their backs, and infected IVAG with a lethal dose of HSV-2 in 10 µl of PBS for at least 45 min while being maintained under anaesthesia. Vaginal washes were collected daily after infection (days 1–3) by pipetting 2×30 µL of PBS into and out of the vagina 6–8 times. Viral titers in IVAG washes were determined by plaque assay on monolayers of Vero cells as previously described [49]. Treated mice were also monitored daily for genital pathology and survival for up to 4 weeks. Pathology was scored on a five point scale. Zero indicated no infection; 1, slight redness of external vagina; 2, swelling and redness of external vagina; 3, severe swelling of external vagina and hair loss in the surrounding area; 4, ulceration of vaginal tissue, redness and swelling; 5, continued ulceration, redness, swelling and sometimes paralysis in back legs, at which point the mice were euthanized.

### Histomorphology of the genital tract

To study the effects of FimH or LPS on vaginal tissue morphology, progesterone-treated mice received FimH (40 µg/mouse) or LPS (5 µg/mouse). After 24 h, vaginal tissue was removed, fixed in 4% paraformaldehyde, embedded in paraffin, and sectioned at 5 µm for hematoxylin and eosin staining.

### ELISA for IFN-α, IFN-β, TNF-α, IFN-γ and IL-8

IFN-α, IFN-β, IFN-γ and IL-8 ELISAs were conducted using Quantikine Murine Kits from R&D Systems (Minneapolis, MN, USA) according to the manufacturer's instructions. IFN-α and IFN-β ELISAs were conducted using PBL Biomedical kits from PBL (Piscataway, NJ, USA). The IFN-α ELISA kit detects mouse IFN-αA, IFN-α1, IFN-α4, IFN-α5, IFN-α6, and IFN-α9, with a detection limit of 10 pg/ml.

### Bacteria and experimental urinary tract infection

A human cystitis isolate of uropathogenic *Escherichia coli* was used for experimental urinary tract infection of mice. *E. coli* NU14-1, which does not express FimH due to a disruption of the *fimH* gene, and *E. coli* NU14, which is the isogenic wild type parent strain, were kindly provided by Dr. Scott Hultgren (Washington University, St. Louis, MO). *E. coli* were cultured in LB broth with streptomycin at 50 µg ml. For mouse infection studies, bacteria were grown overnight in LB broth, washed in 0.85% saline, and resuspended in saline to a concentration of ∼10^9^ colony forming units (cfu) per ml. B6 mice and TLR4^−/−^ were infected with 0.1 ml (10^8^ cfu) of bacterial suspension. A soft catheter (0.7 mm) placed in the urethra of anaesthetized mice and the bacterial were delivered into balder. Urine was collected 0, 2, 6 and 24 hours post-infection and polymorphonuclear leukocytes were quantified using a haemocytometer. Twenty-four hours after infection, the bladders were removed, homogenized and the bacterial load was enumerated.

### Statistical analysis

Statistical differences among the viral titers were determined by analysis of variance followed by Tukey's test. The statistical significances of the survival rates and the percentage of GFP expressing cells were determined by the χ^2^ test. A *P* value of <0.05 was considered statistically significant. An unpaired *t* test was used to determine significant differences in cytokine production.

## Supporting Information

Figure S1 Supplementary Data
(4.22 MB TIF)
