# Supplementary figures and images for: FimH Adhesin of Type 1 Fimbriae Is a Potent Inducer of Innate Antimicrobial Responses Which Requires TLR4 and Type 1 Interferon Signalling
Source: PLoS Pathog. 2008 Dec 5;4(12):e1000233. doi: 10.1371/journal.ppat.1000233 (PMC2585055; doi:10.1371/journal.ppat.1000233)

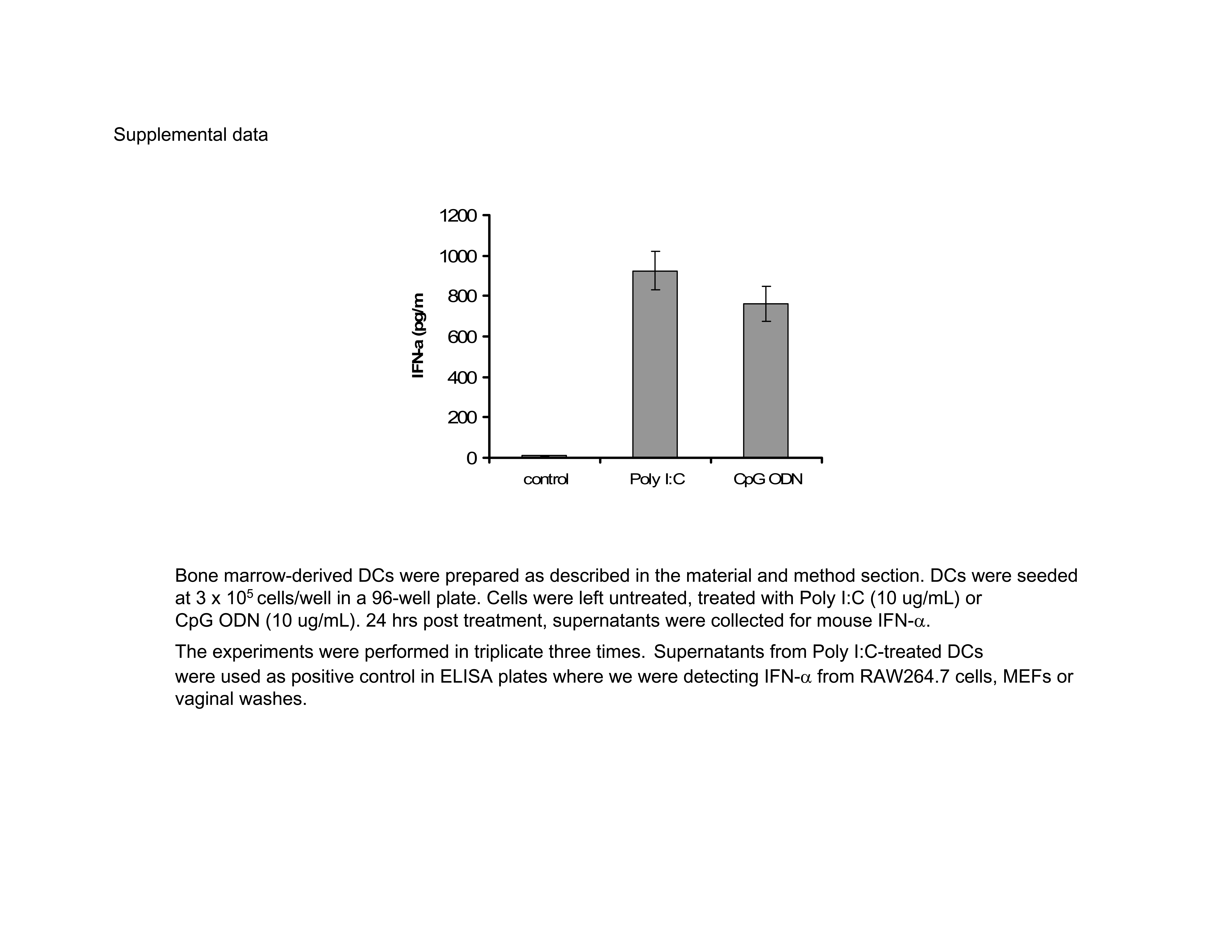

Supplement: Figure S1 — Supplementary Data (4.22 MB TIF) [file ppat.1000233.s001.tif]
